# Novel Absolute Displacement Sensor with Wide Range Based on Malus Law

**DOI:** 10.3390/s91210411

**Published:** 2009-12-21

**Authors:** Wei Li, Xiaoping Lu, Yonggang Lin

**Affiliations:** The State Key Laboratory of Fluid Power Transmission and Control, Zhejiang University, Hangzhou 310027, China; E-Mails: liw@zju.edu.cn (W.L.); lovexiaoping@gmail.com (X.L.)

**Keywords:** displacement sensor, wide range, polarized light, light source drift, absolute measurement

## Abstract

The paper presents a novel wide range absolute displacement sensor based on polarized light detection principle. The sensor comprises of two sets of polarized light detecting systems which are coupled by pulleys. The inherent disadvantage in optic system like light source intensity drift is solved and absolute measurement with wide-range is achieved. A prototype and the relevant test bed have been built. The test results are in good agreement with expectation. Its measurement range is 540 mm, and its linearity is better than 0.05%.

## 1. Introduction

Displacement sensors are widely used in scientific research and industrial processes. With the demand for sensors with wide range growth, new types of sensors have been developed, such as linear induction sensor, magnetic grid sensor and laser sensor. However, most of the sensors have drawbacks such as complicated structure, high cost and low accuracy [1]. To achieve wide range measurement with high accuracy and low cost, many new technologies have been applied in displacement measuring technology [2-4].

The discovery of Malus law in 1809 revealed the method for obtaining and detecting polarized light [5]. Afterwards, researchers have continuously attempted to realize physical measurements with polarized light for, e.g., electric or magnetic field intensity, electric current or voltage, temperature, linear acceleration, angular and linear velocities, force, torque, pressure, *etc.* [6-12]. In 1998, a linear displacement sensor based on Faraday optical rotation was presented by Villaverde [13]. In the same year, Li [14] proposed a novel displacement sensor with wide range based on polarized light detecting technology. The schematic diagram of the sensor was also detailed, and the test results verified the concept. In 2005, Li presented a compact sensor adopting a magnetic garnet crystal working in a saturation state to overcome the temperature drift of the Verdet constant [15]. And to overcome the disadvantages of optical nonlinearity and light source intensity drift as well as to realize wide range measurement, some special mechanical structures and new technologies have been used in this area [16-18].

The absolute measurement is a critical technology in the displacement sensor area [19]. The demand for absolute displacement sensors grows distinctly, but the most of the absolute displacement sensors (e.g., absolute photoelectric encoder) have disadvantages of low resolution, complicated structure and limited measuring range [20]. In this work, a novel wide-range absolute displacement sensor based on Malus law is presented.

### 2. Principle and Configuration

**Figure 1.**
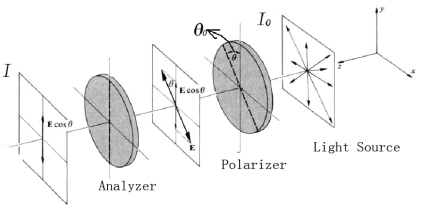
Typical polarized light detecting system.

The working principle of the displacement sensor is based on optical Malus law. In the polarized light detecting system shown in Figure 1, according to optical Malus law, we have [21]:

(1) $$I=I_{0}\cos^{2}\theta_{0}$$

where *I_0_* is the light intensity irradiating from the light source, *I* is the light intensity detected by the analyzer, and *θ*_0_ is the included angle between the polarizer's transmission axes and the analyzer's transmission axes. As long as the light intensity is stable, we can obtain the angular displacement information from the ratio between *I* and *I*_0_. Through an angular displacement-linear displacement convertor, we can get the linear displacement information.

**Figure 2.**
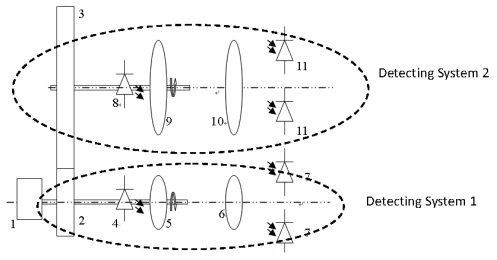
Schematic diagram of the sensor.

In practical measurement, the intensity drift of light source will influence the accuracy of the sensor dramatically. To overcome the drawback of light source drift and realize absolute measurement with wide range, a novel sensor with two polarized light detecting systems is presented. Each system adopts the special structure with one source and multiple light paths. The schematic diagram of the sensor is shown in Figure 2.

The sensor comprises of two individual polarized light detecting systems which are coupled by friction wheels. One named detecting system 1 is used to measure the displacement in one period; the other named detecting system 2 is used to detect the periodic order number *i*, which is the periodic order number of the signal of system 1. As shown in Figure 2, the detecting system 1 consists of wheel 2, LED 4, polarizer 5, analyzer 6 and photodiode group 7. The detecting system 2 consists of wheel 3, LED 8, polarizer 9, analyzer 10 and photodiode group 11. Wheel 2 is coupled with wheel 3 utilizing engagement or rubbing effect, and is coaxially mounted to unit 1, which is an angular displacement-linear displacement convertor.

**Figure 3.**
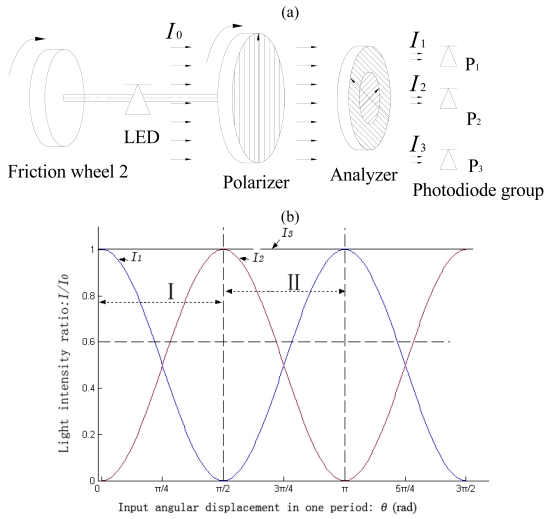
(a) Structure diagram of detecting system 1. (b) Light intensity signals variation rule detected by the detecting system 1.

As shown in Figure 3(a), the analyzer in the detecting system 1 consists of two polarizing discs, which are mounted with their transmission axes being perpendicular. The polarizer is coaxially mounted to the friction wheel 2, so it can trace angular movement of the wheel. The photodiodes group consists of three photodiodes, named P_1_, P_2_ and P_3_. P_1_ and P_2_ respectively detect the intensity of light beams *I*_1_ and *I*_2_, which separately pass through the two polarizing discs mounted in the analyzer, while P_3_ directly detects the light intensity from light source *I*_0_. In the process of the initial system assembly, the established angle between polarizer and analyzer is adjusted to make sure that *I*_1_ is maximized and *I*_2_ is minimized. Upon angular displacement, the light intensity received by the photodiode group will vary regularly as shown in Figure 3(b).

As shown in Figure 3(b), *I*_1_ and *I*_2_ have the same period *π*. To achieve absolute measurement, we divide one period into two equal zones: zone I, and zone II. In zone I, *I*_1_ is selected to be displacement information, whereas in zone II *I*_2_ is chosen to be displacement information. In each part, the light intensity value is unique to any identical point. Which signal should be taken as displacement information is decided by the parity of the periodic order number *i*. Because the analyzer is immobile, the input angular displacement *θ*, which is equal to the included angle *θ*_0_ between transmission axes of the analyzer and the polarizer, will not change even if the power is off. As a result, the absolute measurement in one period can be achieved.

We can get the value of the input angular displacement *θ* from Equation (2). The value of *I* is defined in Equation (3), in which *i* is the periodic order number given by detecting system 2. *I*_0_, *I*_1_ and *I*_2_ are light intensities from the same light source, so the intensity drift of the light source can be eliminated. We can get value of the input linear displacement *S*_1_ from Equation (4), where *k* is the transmission ratio of angular displacement-linear displacement convertor.

(2) $$\theta =\text{arccos}(\sqrt{\frac{I}{I_{0}}})\;(0≦\theta ≦\pi /2)$$

(3) $$I=\{\begin{array}{cc} I_{1} & \text{i is even}\\ I_{2} & \text{i is odd} \end{array}$$

(4) $$S_{1}=k\theta$$

**Figure 4.**
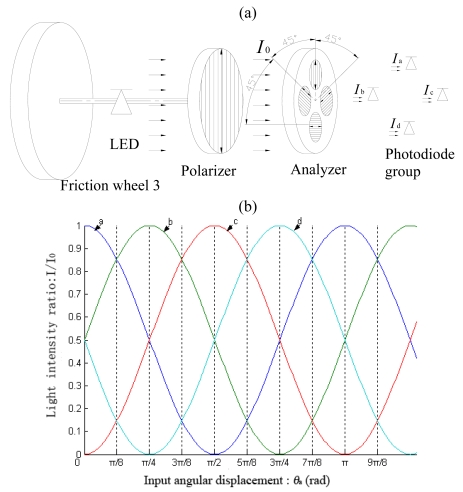
(a) Structure diagram of the detecting system 2. (b) Variation rule of the light intensity signals detected by the detecting system 2.

In the detecting system 2, the polarizer that consists of one polarizing disc is mounted coaxially to friction wheel 3, while the analyzer is fixed and cannot rotate. The number of discs that are mounted in the analyzer of system 2 is equal to *j*, which is the transmission ratio of wheel 2 and wheel 3, and the quantity of photodiodes in photodiode group is equal to *j* as well. The photodiode group is mounted onto the light-back side of the analyzer, and each photodiode detects the light beams passing from the discs in analyzer independently. The polarizing discs are mounted into the analyzer orderly, and the differential angle of the transmission axes of the two adjacent discs is *π/j*. Figure 4(a) shows the diagram of the detecting system 2 when the transmission ratio of the two friction wheels is 4. We set *I*_a_, *I*_b_, *I*_c_, *I*_d_ as the symbols of the signals from the four photodiodes. The variation rule of the signals is shown in Figure 4(b). The information of the periodic order number *i* can be obtained by comparing the relative strength of the four signals. We can get the value of *i* according to the discriminants in Table 1. Because the order number *i* is only related to the relative displacement between polarizer and analyzer, absolute detection of periodic order number can be realized.

The final output of the sensor, which comprises of angular displacement *θ* in one period and periodic order number *i*, can be expressed as follow:

(5) S=(i−1)kπ/2+kθ

And, as *θ* and *i* are related only to the input displacement, the absolute measurement can be achieved.

When the polarizer of system 1 turns for one period (*π*/2), the polarizer of system 2 will turn *π*/2*j*. Because the optical period of sensor system is *π*, we can see the value of the maximum periodic number *i*_max_ is 2*j*, and then the maximum measuring range of the sensor can be expressed as follow:

(6) $$S_{\max}=k\pi i_{\max}/2$$

From Equation 5, we can see the sensor's resolution is determined by system 1 and the measuring range is determined by system 2, and from Equation 6, we can see the measuring range of the sensor can be increased by increasing the transmission ratio of the two wheels. Therefore, if the system 1 remains unchanged, wide range measuring with high resolution can be achieved by increasing the transmission ratio *j* of the two friction wheels.

It is noteworthy that when the transmission ratio *j* is not an integer, the measuring range can be altered by changing the amount of the discs mounted in the analyzer of detecting system 2 without changing the transmission ratio *j*, which means no extra modification of the mechanical structure is required. The symbol *n* is used to represent the quantity of the discs mounted in the analyzer of detecting system 2, and then the value of the maximum periodic number *i*_max_ when *j* is not an integer can be computed by equation: *i*_max_ = 2 × (*n* − 1), herein *n* < *j*. The sensor system with the transmission ratio *j* = 130 / 18 is taken as an example. When n = 4, *i*_max_ = 2 × (4 − 1) = 6, while when n = 8, *i*_max_ = 2 × (8 − 1) = 14.

## 3. Prototype and Experiment

**Figure 5.**
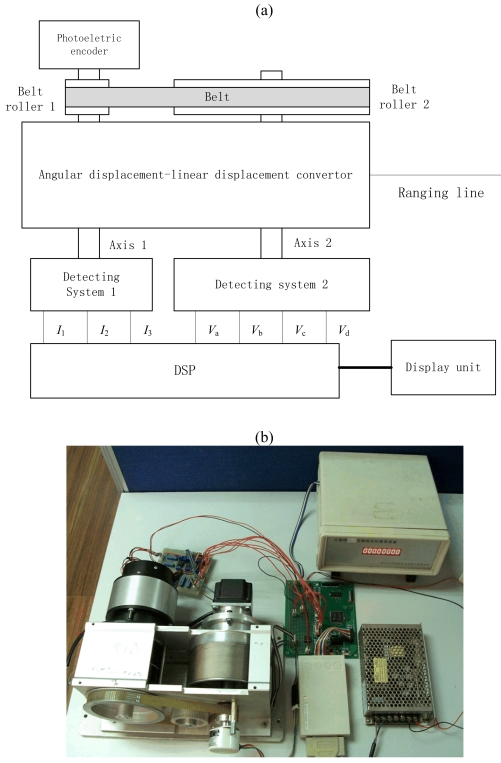

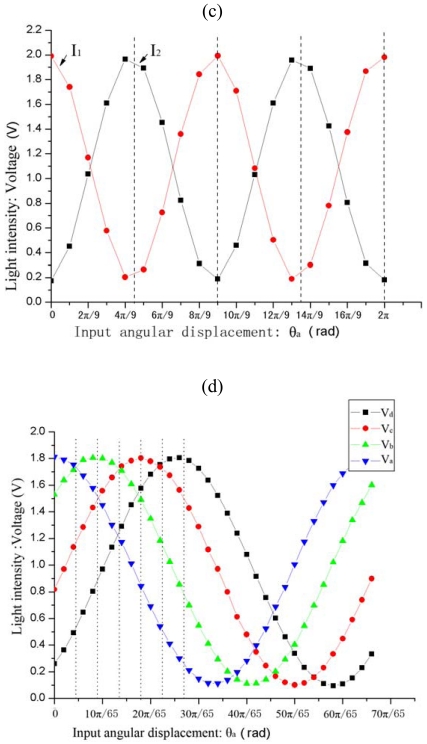
(a) Installation diagram of the prototype and test bed. (b) Entity of the prototype and the test bed. (c) Measured signals of light intensity from detecting system 1 as a response of the input angular displacement. (d) Measured signals of light intensity from detecting system 2 as a response of the input angular displacement.

To verify the concept and test the performance of the sensor, a prototype sensor and its test bed have been fabricated. As shown in Figure 5(a), the test system comprises of the angular displacement-linear displacement convertor, the detecting system 1 for detecting displacement information in one period, the detecting system 2 for detecting the order number of the period, the circuit and software based on DSP, the referenced photoelectric encoder, the and display unit. The transmission ratio of belt roller 1 and 2 is 130/18. The detecting system 1 and referenced photoelectric encoder are both coaxially mounted to belt roller 1, while the detecting system 2 is coaxially mounted to belt roller 2. There are four discs mounted in the analyzer of the detecting system 2, which means there are four lead-out signals from detecting system 2. The measured voltage signals' variation rule is shown in Figure 5(c), (d). The order number of period can be discriminated according to the Table 2. The value of maximum periodic order number *i*_max_ is 6 as computed in the last section.

The transmission and extinction coefficient of polarizers cannot reach 100%, so the light intensity cannot reach 0 when the polarizers are crossed or reach 1 when the polarizers are parallel. Segment fitting is processed by the software in the DSP to overcome the nonlinearity and get the position information in one period. The resolution of the sensor system is related to the fitting degree of the software and independent of the measuring range. The DSP adopts NI's product TMS320LF2407 whose A/D conversion digit number is 10. As the period of detecting system 1 is π/2, the resolution of the sensor can be computed as this: (*π*/2)/2^10^ = *π*/2048.

The angular displacement-linear displacement convertor consists of a high-accuracy wire spool convertor, a shrinker with constant force and a screw displacement compensator. The transmission ratio *k* of the convertor is 180/*π* (mm/rad), so the linear resolution of the sensor is:

(π/2048)×(180/π)=180/2048=0.09mm

The maximum measuring range *S_max_* of the sensor can be computed as follow:

$$S_{\max}=i_{\max}k\pi /2=6\times 180\times \pi /(2\pi )=540mm$$

The incremental photoelectric encoder IE40-L3600_3 which is produced by ASM Company is taken as referenced displacement sensor. The photoelectric encoder has the measuring range up to 3,600 mm, while its linearity is 0.01%. The resolution of the encoder is *π*/7200, and the linear resolution is: (*π*/7200) × (180/*π*) = 0.025 mm.

**Figure 6.**
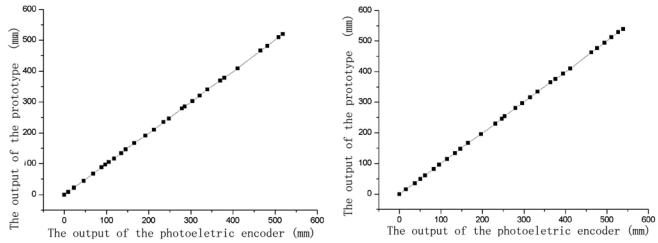
(a) Comparison between the positive measuring output of the sensor and referenced displacement. (b) Comparison between the negative measuring output of the sensor and referenced displacement.

The experiments have been carried out to test the performance of the prototype sensor. Figure 6 shows the comparison between the positive and negative measuring output of the sensor and referenced displacement sensor. The results show that the measuring range of the sensor can reach the expected value 540 mm. After fitting the curve, we can see the sensor has the linearity better than 0.05%, and its error range is less than 0.2 mm.

The repeatability experimental process is as follow: First, draw the ranging line to three set points, record the measured value, and then let the ranging line return to the initial point, after that pull the ranging line to the points again. Six groups of data have been recorded as shown in Table 3. The results show that the sensor has good repeatability with the repeatability error less than 0.1 mm.

**Figure 7.**
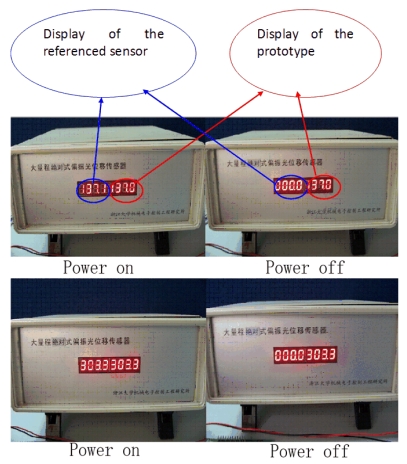
Absolute measuring ability test when there is no extra displacement is generated.

To verify the absolute measuring ability, the test of absolute measuring ability has been carried out. The process and the results are as follow:

1. No extra displacement is generated when power supply is off. First, draw the ranging line to a set point, record the output of the sensor, after that cut off the current supply, then turn on the supply after a few minutes, and record the new output. While the power is off, the ranging line is kept at the set point. The results are shown in Figure 7.
2. Extra displacement is generated when power supply is off. First, mark several position points, record their displacement values, after that cut off the power supply, and pull the ranging line to the marked points from random position, and then turn on the supply, record the new display of the sensor. The results are shown in Table 4.

The experiment shows that the output of sensor is unique to any identical point in its measuring range, and is independent to the status of the power supply, which means the sensor is capable of making absolute measurement.

## 4. Conclusions

A novel type of absolute displacement with wide range based on Malus law has been developed, and a prototype has been fabricated. The experimental results show that the sensor has the linearity better than 0.05%, while its measuring range is up to 540 mm. The results also verify absolute measurement ability of the sensor. Comparing with the absolute encoder, the sensor can realize wide range measurement with high resolution. The sensor has additional merits such as simple structure and low cost.

## Figures and Tables

**Table 1.** The discriminant of periodical information.

Periodical order number *i*	Discriminant
1	I_a_ > I_b_ ≥ I_d_ > I_c_
2	I_b_ ≥ I_a_ > I_c_ ≥ I_d_
3	I_b_ > I_c_ ≥ I_a_ > I_d_
4	I_c_ ≥ I_b_ > I_d_ ≥ I_a_
5	I_c_ > I_d_ ≥ I_b_ > I_a_
6	I_d_ ≥ I_c_ > I_a_ ≥ I_b_
7	I_d_ > I_a_ ≥ I_c_ > I_b_
8	I_a_ ≥ I_d_ > I_b_ ≥ I_c_

**Table 2.** The discriminant of period order number information.

Periodical order number i	Discriminant
1	V_a_ > V_b_ > V_c_ > V_d_
2	V_b_ ≥ V_a_ > V_c_ > V_d_
3	V_b_ > V_c_ ≥ V_a_ > V_d_
4	V_c_ ≥ V_b_ > V_d_ ≥ V_a_
5	V_c_ > V_d_ ≥ V_b_ > V_a_
6	V_d_ ≥ V_c_ > V_b_ > V_a_

**Table 3.** Repeatability experiment data.

Point 1 (mm)	No.	Output (mm)	Point 2 (mm)	No.	Output (mm)	Point 3 (mm)	No.	Output (mm)
110.0	1	110.5	240.0	1	240.4	480.0	1	480.2
	2	110.5		2	240.4		2	480.2
	3	110.6		3	240.4		3	480.2
	4	110.5		4	240.5		4	480.2
	5	110.5		5	240.4		5	480.2
	6	110.5		6	240.5		6	480.2
Repeatability error (mm)		0.1	Repeatability error (mm)		0.1	Repeatability error (mm)		0.0

**Table 4.** Absolute measuring ability test results (extra displacement happens).

**No.**	**Calibration displacement value (mm)**	**Output (mm)**
1	59.8	59.8
2	124.6	124.6
3	191.6	191.6
4	302.7	302.7
5	379.7	379.7
6	509.2	509.2
